# Accuracy of AI chatbots in answering frequently asked questions on cervical cancer

**DOI:** 10.3389/frai.2025.1655303

**Published:** 2025-09-01

**Authors:** Jielin Fan, Wenhong Xiao, Zhipeng Yan, Qiang Ouyang

**Affiliations:** ^1^Department of Gynecologic Oncology, Hunan Cancer Hospital, The Affiliated Cancer Hospital of Xiangya School of Medicine, Central South University, Changsha, China; ^2^Department of Gynecology, The Second Zhuzhou Hospital, Zhuzhou, China

**Keywords:** cervical cancer, frequently asked questions, artificial intelligence, DeepSeek, ChatGPT

## Abstract

**Objective:**

To compare the accuracy of Deepseek and ChatGPT in answering frequently asked questions (FAQs) about cervical cancer.

**Methods:**

To compile a list of FAQs concerning cervical cancer, a comprehensive search was conducted on social media and community platforms. The answer keys for all the selected questions were created on the basis of the guidelines of the National Comprehensive Cancer Network (NCCN), the International Federation of Gynecology and Obstetrics (FIGO), and the World Health Organization (WHO) for cervical cancer. The answers given by Deepseek-R1 and ChatGPT O1 were scored according to the Global Quality Score (GQS).

**Results:**

A total of 74 FAQs covered a diverse range of topics related to cervical cancer, including diagnosis (*n* = 16), risk factors and epidemiology (*n* = 19), treatment (*n* = 20), and prevention (*n* = 19). When all the answers provided by DeepSeek to the FAQs about cervical cancer according to the GQS were evaluated, 68 answers were rated as score five, 4 answers were rated as score four, and 2 answers were rated as score three. For ChatGPT’s responses to the same set of FAQs, 67 answers were classified as score five, 6 answers were classified as score four, and 1 answer was classified as score three. There was no statistically significant difference between the two groups (*p* > 0.05).

**Conclusion:**

Both DeepSeek and ChatGPT demonstrated accurate and satisfactory responses to FAQs about cervical cancer when evaluated according to the GQS. However, in regard to treatment issues, a cautious attitude should be maintained. Compared to ChatGPT, DeepSeek stands out for its free availability, which makes it more accessible in resource-limited scenarios to the public.

## 1 Introduction

Cervical cancer ranks as the fourth most prevalent cancer among women globally. In 2022, approximately 661,021 new cases were diagnosed, leading to 348,189 deaths (Bray et al., 2024). Socioeconomic, racial and ethnic disparities exist with respect to human papillomavirus (HPV) vaccination, cervical cancer screening and cervical cancer survival worldwide and nationally (Francoeur et al., 2025). For example, low-resource regions of Latin America, sub-Saharan Africa, and Southeast Asia, including China, bear a heavy disease burden (Tewari, 2025). Given the long premalignant phase of cervical cancer, this period offers a crucial window for prevention and treatment, making popular science education on cervical cancer essential. The internet has emerged as a powerful tool in this educational effort.

Large language model (LLM)-based chatbots have been publicly available and increasingly utilized by the general population since 2022. LLM-based chatbots are revolutionizing healthcare by leveraging advanced algorithms and data-driven insights to address critical challenges in diagnosis, treatment and patient care. DeepSeek (http://www.deepseek.com), an interactive LLM-based chatbot launched in 2024, has experienced rapid growth in popularity in both public and private domains since its release. Compared to ChatGPT, its free use and publicly available technology have also attracted many users. Semeraro et al. (2025) evaluated the effectiveness of ChatGPT-4o, Gemini Advanced and DeepSeek in disseminating the latest European Resuscitation Council (ERC) 2021 guidelines to the general public. All tools demonstrated strong alignment with the ERC 2021 standards. In China, an increasing number of people are adopting an open attitude toward new technologies, believing that artificial intelligence (AI) can provide more accurate diagnoses and personalized treatment plans, which can help improve their health status. However, some physicians still have certain concerns about the reliability and accuracy of AI technology.

Previous reports have analyzed the knowledge of various medical conditions related to ChatGPT (Yurtcu et al., 2025; Hermann et al., 2023). However, the performance of DeepSeek in medical problems has received only limited coverage in the literature. This study aims to compare the accuracy of DeepSeek and ChatGPT in answering frequently asked questions (FAQs) concerning cervical cancer.

## 2 Materials and methods

### 2.1 Question selection

Since this study lacked patient data, neither informed consent nor ethics committee approval was obtained. The list of questions included common inquiries by the public, and comments about cervical cancer were searched on social media and community platforms. Questions asked for advertising purposes, questions of an unrealistic nature, repetitive inquiries, questions without proper grammatical structure, and questions related to subjective answers and personal health were excluded from the study. In total, 74 questions on the FAQs form were categorized as questions about diagnosis, risk factors and epidemiology, treatment, and prevention.

### 2.2 Evaluation procedure

The answer keys were created for all the questions on the basis of the National Comprehensive Cancer Network (NCCN), International Federation of Gynecology and Obstetrics (FIGO), and World Health Organization (WHO) cervical cancer guidelines. The answer keys were cross-checked by two experienced gynecologists (JLF and ZPY), and any disagreements were resolved through consensus discussions. All the questions were answered by DeepSeek (R1 model) and ChatGPT (O1 model) on the same day. The answers given by DeepSeek and ChatGPT were analyzed by two experienced gynecologists (JLF and ZPY), and the answers were scored according to the Global Quality Score (GQS) (Mangan et al., 2020) (Table 1). The responses of DeepSeek and ChatGPT were scored by evaluating their conformity to this answer key. The answers were rated between 1 and 5 (worst to best), and the quality, accuracy, and comprehensiveness of the information provided were assessed. The specialists had not seen these responses previously, the responses were anonymized (the name of the chatbot was hidden) and the order of responses was randomized to prevent bias during evaluation. If the scores given by the specialists to the answer were different, the answer was reviewed by two specialists, and the score was given by the joint decision of the two specialists.

**Table 1:** Global quality score.

Score	Definition
1	Poor quality, very unlikely to be of any use to patients
2	Poor quality but some information present, of very limited use to patients
3	Suboptimal flow, some information covered but important topics missing, somewhat useful to patients
4	Good quality and flow, most important topics covered, useful to patients
5	Excellent quality and flow, highly useful to patients

### 2.3 Statistical analysis

Statistical analysis were performed via IBM SPSS Statistics 28.0 (IBM Corp., USA). The Wilcoxon signed-rank test was employed to compare the accuracy and comprehensiveness of responses generated by DeepSeek-R1 and ChatGPT O1. The significance of difference between two groups was determined using the two-tailed Student’s *t*-test. Mann–Whitney U-test was used for data that did not conform to a normal distribution. *p* < 0.05 indicated significance.

## 3 Results

### 3.1 Overall results

**Figure 1:**
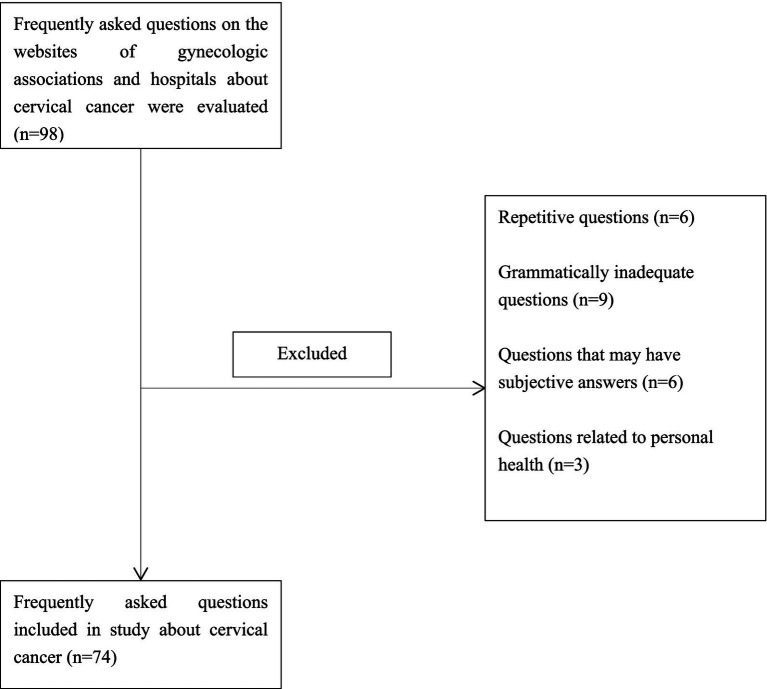
Flowchart for frequently asked questions included in the study.

In total, 98 FAQs were created based on questions on websites of professional health institutes and hospitals, websites frequently used by patients, and social media applications. 6 repetitive questions, 9 questions with inadequate grammar, 6 inquiries with subjective answers, and 3 questions related to personal health were excluded from the study, and the remaining 74 questions were answered by DeepSeek-R1 and ChatGPT O1. The flowchart for the present study is presented in Figure 1. These questions encompass a range of topics, including diagnosis (between the 1st and 16th questions), risk factors and epidomylogy (between the 17th and 35th questions), treatment (between the 36th and 55th questions), and prevention (between the 56th and 74th questions) of cervical cancer (Supplementary Table S1).

When all DeepSeek answers to FAQs about cervical cancer were evaluated with respect to GQS, 68 answers were classified as score five, 4 answers were classified as score four, and 2 answers were classified as score three. When all the ChatGPT answers to the same FAQs about cervical cancer were evaluated with respect to the GQS, 67 answers were classified as score five, 6 answers were classified as score four, and 1 answer was classified as score three. None of the DeepSeek or ChatGPT answers for the FAQs were scored as two or one (Table 2).

**Table 2:** GQS score for answers by DeepSeek and ChatGPT to questions related to cervical cancer.

	GQS score	DeepSeek-R1	ChatGPT O1	*p*-value
FQAs (*n* = 74)	5	68 (91.9%)	67 (90.5%)	0.526
	4	4 (5.4%)	6 (8.1%)	
	3	2 (2.7%)	1 (1.4%)	
	2	-	-	
	1	-	-	
Diagnosis (*n* = 16)	5	16 (100.0%)	15 (93.8%)	0.617
	4	-	1 (6.2%)	
	3	-	-	
	2	-	-	
	1	-	-	
Risk factors and epidemiology (*n* = 19)	5	18 (94.7%)	17 (89.5%)	0.562
	4	1 (5.3%)	2 (10.5%)	
	3	-	-	
	2	-	-	
	1	-	-	
Treatment (*n* = 20)	5	17 (85.0%)	18 (90.0%)	0.375
	4	1 (5.0%)	1 (5.0%)	
	3	2 (10.0%)	1(5.0%)	
	2	-	-	
	1	-	-	
Prevention (*n* = 19)	5	17 (89.5%)	17 (89.5%)	1.000
	4	2 (10.5%)	2 (10.5%)	
	3	-	-	
	2	-	-	
	1	-	-	

**Figure 2:**
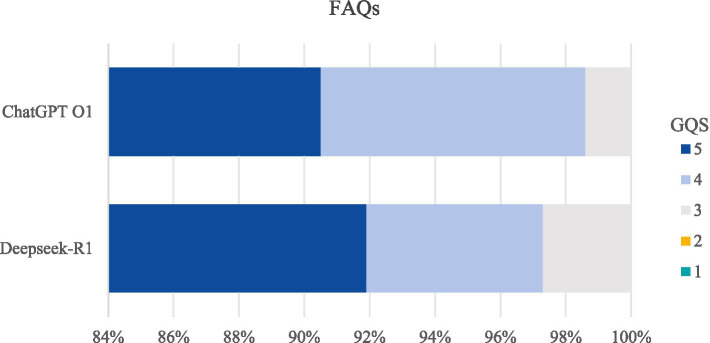
Comparison of GQS score for FAQs between DeepSeek-R1 and ChatGPT O1.

The bar chart illustrated the ratings of responses provided by DeepSeek and ChatGPT, evaluated according to the GQS. The horizontal axis scale (1–100) represents the percentage distribution of scores across different levels on the GQS (Figure 2). The results indicated that there was no statistically significant difference between the two groups (*p* > 0.05). This implies that, on average, both DeepSeek and ChatGPT demonstrated similar levels of performance in answering the 74 cervical cancer-related FAQs in terms of the quality, accuracy, and comprehensiveness of the information provided.

### 3.2 Analysis of subgroup results

In the diagnosis subgroup, DeepSeek demonstrated perfect performance, with 16 out of 16 (100.0%) responses categorized as GQS five. ChatGPT, on the other hand, had 15 out of 16 (93.8%) responses rated as GQS five, and 1 out of 16 (6.2%) responses were classified as GQS four. This finding indicates that DeepSeek was more consistent in providing top-quality answers regarding cervical cancer diagnosis questions.

With respect to the risk factors and epidemiology subgroup, DeepSeek had 18 out of 19 (94.7%) responses rated as GQS five and 1 out of 19 (5.3%) responses rated as GQS four. ChatGPT had 17 out of 19 (89.5%) responses rated as GQS five and 2 out of 19 (10.5%) responses rated as GQS four. Although both models performed well, DeepSeek had a slightly greater proportion of top-rated answers in this subgroup.

In the treatment subgroup, 17 out of 20 (85.0%) DeepSeek responses were categorized as GQS five, 1 out of 20 (5.0%) as GQS four, and 2 out of 20 (10.0%) as GQS three. ChatGPT had 18 out of 20 (90.0%) responses rated as GQS five, 1 out of 20 (5.0%) as GQS four, and 1 out of 20 (5.0%) as GQS three. Here, ChatGPT had marginally better performance in terms of the proportion of GQS five answers, but the differences were relatively small.

For the prevention subgroup, both DeepSeek and ChatGPT showed similar performance. 17 out of 19 (89.5%) responses from both chatbots were rated as GQS five, and 2 out of 19 (10.5%) responses were classified as GQS four. This suggests that in answering questions related to cervical cancer prevention, the two models were equally competent in providing high-quality information.

**Figure 3:**
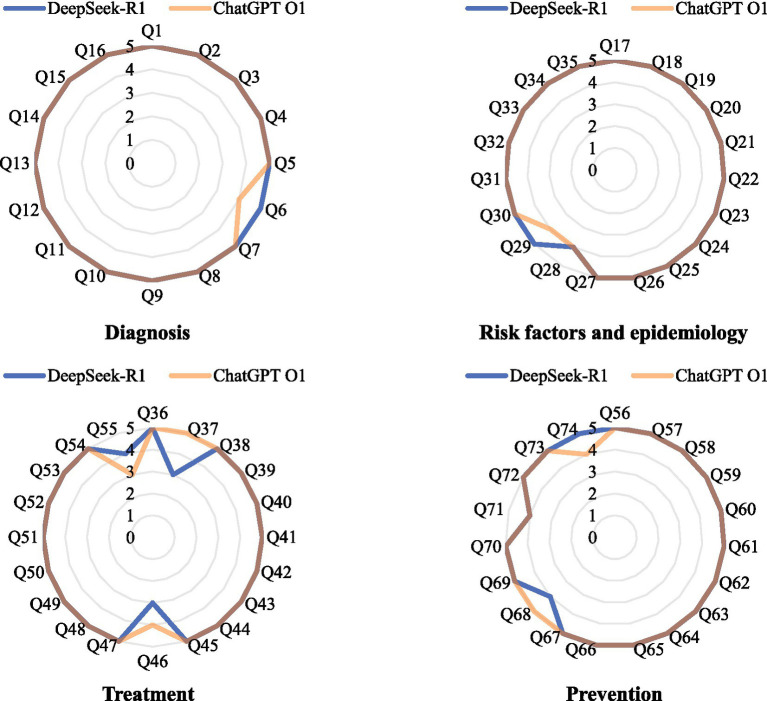
Performance of DeepSeek-R1 (blue) and ChatGPT O1 (orange) in four subgroups.

The radar chart showed the performance of DeepSeek-R1 and ChatGPT O1 in the diagnosis, risk factors and epidemiology, treatment, and prevention subgroup (Figure 3). The result of statistical analysis showed that there was no statistically significant difference between the two groups (*p* > 0.05).

## 4 Discussion

Cervical cancer is the only malignant tumor for which the cause is known, with more than 90% of cases linked to HPV infection. The long premalignant phase of cervical cancer and the natural progression of the disease make cervical cancer the only cancer that is currently preventable through primary and secondary prevention (Wu et al., 2024). Artificial intelligence (AI), particularly through large language models such as DeepSeek and ChatGPT, can help physicians and patients make informed decisions regarding the selection of evidence-based treatment plans tailored to the specific needs of patients. However, it is critical to remain cautious, as these LLM-based chatbots, despite their capabilities, might still deliver inaccurate information (Mija et al., 2024). In this study, we compared DeepSeek and ChatGPT in terms of their ability to answer FAQs for the treatment of cervical cancer and assessed their accuracy in agreement with the NCCN, FIGO, and WHO recommendations. The answers were scored according to the GQS.

Despite LLM-based chatbots demonstrating immense potential in the medical field, they still face numerous technical hurdles in practical applications. The quality of data is a pivotal factor influencing the efficacy of medical applications. Hermann et al. (2023) quantified the accuracy of ChatGPT responses to cervical cancer questions. The results revealed that ChatGPT performed less well in the treatment category, with 15/21 (71.4%) correct scores, and performed the worst in the diagnosis category, with only 1/3 (33.3%) correct scores. Yurtcu et al. (2025) reported that ChatGPT gave complete accurate and satisfactory responses to 91.9% of FAQs, but the accuracy rate decreased to 62.3% when questions were answered on the basis of the European Society of Gynecological Oncology (ESGO), European Society for Radiotherapy and Oncology (ESTRO), and European Society of Pathology (ESP) guidelines for cervical cancer. Our results revealed that 2 of 20 (10.0%) DeepSeek responses and 1 of 20 (5.0%) ChatGPT responses were categorized as GQS three in the treatment subgroup. In other subgroups, including diagnosis, risk factor and epidomylogy and prevention of cervical cancer, the answers of DeepSeek were all categorized as GQS four or five.

In our study, there was no statistically significant difference in the average GQS score between DeepSeek and ChatGPT in answering frequently asked questions about cervical cancer. However, Gianluca et al. (2025) compared the performance of the ChatGPT O1 and DeepSeek-R1 models on a sample of pediatric questions drawn from the MedQA dataset. The results revealed that ChatGPT achieved an accuracy of 92.8%, while DeepSeek achieved an accuracy of 87.0%, and the differences in the classification patterns of the two models were statistically significant. ChatGPT O1 employs “chain-of-thought reasoning” to enhance structured problem-solving, whereas DeepSeek-R1 introduces self-reflection capabilities through reinforcement learning and achieves high performance at a fraction of ChatGPT’s cost (training costs 58 million vs. ChatGPT’s 320 million). The low cost of DeepSeek makes it particularly useful in resource-limited healthcare environments or academic projects that require free and flexible tools.

This study has several limitations. First, the sample size was limited, including only a small number of FAQs and focusing on only two chatbots, DeepSeek and ChatGPT. The FAQs were also restricted to the English language, which may limit the generalizability of the findings (Luo et al., 2025). Second, the responses of the AI chatbots were based on the information available on websites up to the study date. Given the dynamic nature of medical knowledge and the continuous emergence of new research findings, some answers may quickly become outdated. Additionally, the answers to certain questions can be controversial across different medical studies and guidelines; however, this study only evaluated responses on the basis of specific guidelines, potentially overlooking other valid perspectives (Eralp and Sefer, 2024). Finally, the accuracy of the answers was solely evaluated by physicians according to the GQS. The evaluation did not consider the understandability and usefulness of the chatbots’ suggestions from the public’s perspective.

For future research, a larger and more diverse sample of FAQs, including those in multiple languages, should be examined to provide a more comprehensive assessment of the performance of AI chatbots in answering cervical cancer-related questions. To address the issue of data timeliness, continuous monitoring and updating of the information sources used by chatbots, as well as regular re-evaluations of their responses, are recommended. The incorporation of multiple guidelines and a broader range of medical literature in the evaluation process could offer a more balanced view of chatbot accuracy. Moreover, future studies could focus on evaluating the understandability and practical usefulness of AI chatbots’ suggestions to the public, perhaps through surveys or user-feedback mechanisms. This would provide a more holistic understanding of the role and potential of these chatbots in cervical cancer patient education and healthcare decision-making.

In conclusion, DeepSeek and ChatCPT both showed accurate and satisfactory responses to FAQs about cervical cancer with respect to the GQS. However, in regard to treatment issues, a cautious attitude should be maintained. Compared with ChatGPT O1, DeepSeek-R1 stands out for its free availability, which makes it more accessible in resource-limited scenarios to the public.

## Data Availability

The original contributions presented in the study are included in the article/Supplementary material, further inquiries can be directed to the corresponding author.
