# Supplementary material for: Accuracy of AI chatbots in answering frequently asked questions on cervical cancer
Source: Front Artif Intell. 2025 Sep 1;8:1655303. doi: 10.3389/frai.2025.1655303 (PMC12433935; doi:10.3389/frai.2025.1655303)
Supplement: Supplementary file 1 [file Table_1.DOCX]

**Table S1** Frequently asked questions related cervical cancer

| **Topics** | **Frequently Asked Questions (FAQs)** |
| --- | --- |
| Diagnosis | 1-What is cervical cancer, and how does it develop? |
|  | 2-What are the common symptoms of cervical cancer that may lead to diagnosis? |
|  | 3-How is cervical cancer diagnosed? |
|  | 4-What is a Pap smear, and how is it used in cervical cancer diagnosis? |
|  | 5-When should women start getting regular Pap smears? |
|  | 6-Are there any other screening tests for cervical cancer besides Pap smears? |
|  | 7-What is HPV, and how is it related to cervical cancer diagnosis? |
|  | 8-Can cervical cancer be diagnosed without the presence of HPV? |
|  | 9-Is a colposcopy a common part of cervical cancer diagnosis? |
|  | 10-What does a biopsy involve, and when is it performed in cervical cancer diagnosis? |
|  | 11-How long does it typically take to receive the results of a cervical biopsy? |
|  | 12-Are there any imaging tests used in the diagnosis of cervical cancer? |
|  | 13-What does a positive cervical cancer diagnosis mean, and how is it confirmed? |
|  | 14-Can cervical cancer be diagnosed at an early stage, and why is early detection important? |
|  | 15-What are the different stages of cervical cancer, and how is stage determined? |
|  | 16-Can cervical cancer be diagnosed during pregnancy, and how is it managed in such cases? |
| Risk factors and epidemiology | 17-How can I reduce my risk of developing cervical cancer and the need for a diagnosis? |
|  | 18-What are the primary risk factors for cervical cancer? |
|  | 19-How common is cervical cancer worldwide? |
|  | 20-Are there geographic variations in cervical cancer rates? |
|  | 21-What is the role of human papillomavirus (HPV) in cervical cancer risk? |
|  | 22-Can HPV infection be prevented, and does it always lead to cervical cancer? |
|  | 23-How does smoking tobacco affect the risk of developing cervical cancer? |
|  | 24-Are there any genetic factors that contribute to cervical cancer risk? |
|  | 25-Does a family history of cervical cancer increase one's risk? |
|  | 26-How does age affect the likelihood of developing cervical cancer? |
|  | 27-Is there a connection between sexual activity and cervical cancer risk? |
|  | 28-Can the use of birth control methods impact cervical cancer risk? |
|  | 29-What is the relationship between cervical cancer and pregnancy? |
|  | 30-Does a weakened immune system increase the risk of cervical cancer? |
|  | 31-Are there any dietary factors that may influence cervical cancer risk? |
|  | 32-Is there a correlation between certain sexually transmitted infections (STIs) and cervical cancer risk? |
|  | 33-Are there disparities in cervical cancer risk among different racial or ethnic groups? |
|  | 34-Can vaccination against HPV help reduce the risk of cervical cancer? |
|  | 35-What strategies are in place to address and reduce cervical cancer risk globally? |
| Treatment | 36-What are the treatment options available for cervical cancer? |
|  | 37-How is the choice of treatment determined for an individual with cervical cancer? |
|  | 38-What is the standard treatment for early-stage cervical cancer? |
|  | 39-Is surgery a common treatment option for cervical cancer, and what types of surgeries are performed? |
|  | 40-What is radiation therapy, and how is it used in cervical cancer treatment? |
|  | 41-Are chemotherapy and immunotherapy used in the treatment of cervical cancer? |
|  | 42-How is the stage of cervical cancer determined, and why is it important for treatment decisions? |
|  | 43-Can fertility-sparing treatments be considered for young women with cervical cancer who wish to have children? |
|  | 44-What are the potential side effects of cervical cancer treatments, and how are they managed? |
|  | 45-Is there a role for targeted therapy in cervical cancer treatment? |
|  | 46-Are clinical trials an option for cervical cancer patients, and how can one participate in them? |
|  | 47-What is the role of palliative care in cervical cancer treatment, and when is it considered? |
|  | 48-How often should cervical cancer patients have follow-up appointments after completing treatment? |
|  | 49-Are there complementary or alternative therapies that can be used alongside conventional treatments? |
|  | 50-Can cervical cancer recur after treatment, and how is recurrence monitored and managed? |
|  | 51-Are there specific dietary or lifestyle recommendations for cervical cancer patients during and after treatment? |
|  | 52-How does the treatment of advanced or metastatic cervical cancer differ from early-stage cancer? |
|  | 53-Are there long-term effects or complications of cervical cancer treatment to be aware of? |
|  | 54-What resources and support are available to help patients cope with the emotional and practical aspects of treatment? |
|  | 55-How can I be proactive in managing my treatment and recovery from cervical cancer? |
| Prevention | 56-What can I do to prevent cervical cancer? |
|  | 57-Is there a vaccine available to prevent cervical cancer, and who should get it? |
|  | 58-How does the HPV vaccine work in preventing cervical cancer? |
|  | 59-When should I or my child receive the HPV vaccine? |
|  | 60-Is it still necessary to get regular Pap smears if I've been vaccinated against HPV? |
|  | 61-Can I get the HPV vaccine if I'm already sexually active or older? |
|  | 62-Are there any side effects or risks associated with the HPV vaccine? |
|  | 63-How can I reduce my risk of contracting HPV, aside from vaccination? |
|  | 64-Is safe sex an effective way to prevent cervical cancer and HPV infection? |
|  | 65-How often should I undergo cervical cancer screening, and when should I start? |
|  | 66-What is the recommended age to begin cervical cancer screening? |
|  | 67-Is it necessary to continue screening after menopause? |
|  | 68-Are there alternative screening methods to Pap smears, such as HPV testing? |
|  | 69-Can smoking cessation help in preventing cervical cancer? |
|  | 70-Does a healthy diet and lifestyle play a role in cervical cancer prevention? |
|  | 71-What is the relationship between birth control methods and cervical cancer prevention? |
|  | 72-Are there any specific preventive measures for individuals with a family history of cervical cancer? |
|  | 73-Can regular exercise help reduce the risk of cervical cancer? |
|  | 74-Where can I find reliable information and resources on cervical cancer prevention? |
